# Follow or Question? Hidden Diversity and Miscellaneous Thought on the Subspecific Classification of *Marchantia emarginata* Reinw., Blume & Nees (Marchantiaceae, Marchantiophyta) Complex

**DOI:** 10.1002/ece3.70714

**Published:** 2024-12-23

**Authors:** Tian‐Xiong Zheng

**Affiliations:** ^1^ Hattori Botanical Laboratory Nichinan Japan

**Keywords:** history, *M. emarginata* complex, *Marchantia*, specimen examination, taxonomic review

## Abstract

*Marchantia emarginata* Reinw., Blume & Nees, with nearly 30 assigned names, is considered the most taxonomically complex species in the family Marchantiaceae. Currently, this species is segregated into three subspecies, and this subspecific classification is widely accepted since its formal inception. However, due to its extensive morphological variation and ambiguous intraspecific delimitation, many bryologists struggle to accurately identify this species at a subspecific level. Through scrutiny of related literatures and morphological examination of over 200 herbarium specimens, the taxonomic history, issues, and various perspectives on this species were newly summarized. Each subspecies was found to exhibit excessive morphological diversity. Consequently, the prevalent subspecific classification of *M. emarginata* was partly challenged by the morphological evidence obtained in the present study. This species urgently requires taxonomic revision using an integrative approach.

## Graphical abstract

I focused on a taxonomic difficulty in liverwort taxonomy, *Marchantia emarginata* , and creatively summarized its history, issues, and miscellaneous thought through my literature scrutiny and morphological examination of over 200 herbarium specimens, in order to provide a reliable reference for a better understanding of the taxonomy of a model plants system Marchantiaceae.

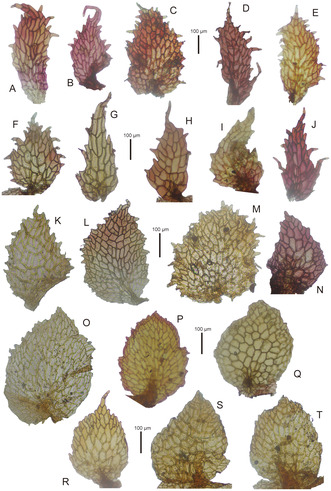

## 1 Introduction

**FIGURE 1:**
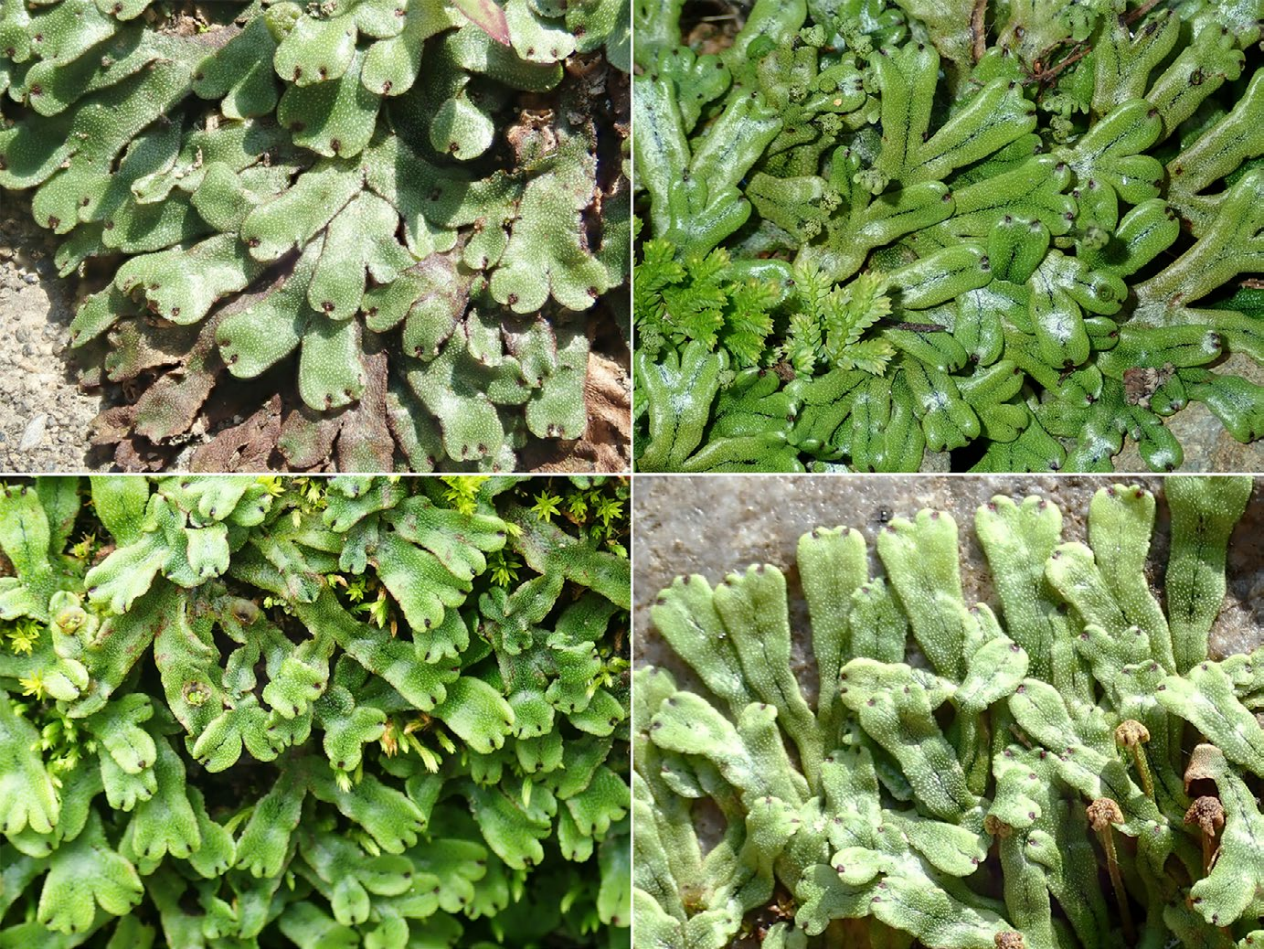
*Marchantia emarginata* Reinw., Blume & Nees *sensu* Bischler (1989) in Japan.

Recent publications on the taxonomy of Marchantiaceae, including novel morphological characters (Zheng and Shimamura 2019; Zheng, Inoue, and Shimamura 2020; Zheng 2023), regional records (Zheng and Shimamura 2020; Zheng, Long, and Shimamura 2023), nomenclatural issues (Zheng 2021; Zheng et al. 2024a), and new perspectives (Long et al. 2016; Zheng and Shimamura 2022a; Zheng et al. 2024b), have advanced the knowledge in terms of this family to a new level since the serial monographic studies published around 40 years ago (Bischler 1984; Bischler‐Causse 1989, 1993). However, as for the previous treatment of the sect. *Papillatae* Bischl., it has not been discussed in detail or updated significantly, likely due to its ambiguous inter‐ and intra‐specific delimitation (Bischler 1989), resulting in broad morphological plasticity and high taxonomic complexity. As a member of this section, *Marchantia emarginata* Reinw., Blume & Nees is widely distributed in Asia and Oceania and has been assigned a broad species definition (Figure 1).

During an integrative study on the family Marchantiaceae, I noticed morphological incongruencies among subsp. *emarginata* and subsp. *lecordiana* (Steph.) Bischl. of *Marchantia emarginata* . In other words, the two subspecies and current subspecific treatment of this species is partly challenged by morphological evidence. Here, I conducted a literature review and specimen examination, and provided the following notes on *Marchantia emarginata* , to serve as a reference for future comprehensive revisions of the sect. *Papillatae*. In the present study, no formal taxonomic treatment was proposed.

## 2 Materials and Methods

Given that subsp. *emarginata* and subsp. *lecordiana* of *Marchantia emarginata* are widely distributed in Oceania and Southeast Asia, the specimens examined in this study (including several types) were mainly borrowed from herbaria that preserve a large number of such samples (CANB, CBG, G, JE, NICH, NY, and TNS), supplemented by some specimens from East Asia (HIRO, IFP, and PE). These herbaria have also been cited several times in series of studies on the taxonomy of *M. emarginata* (Bischler 1989; Bischler‐Causse 1989). Ordinary specimens of *Marchantia emaginata* subsp. *emarginata* and subsp. *lecordiana* identified by Bischler were the primary focus to better understand the original conception of Bischler (1989) and Bischler‐Causse (1989). In this study, specimens identified by Bischler (Bischler‐Causse) are marked with an asterisk (*) at the end of the herbarium code (Appendix A).

Examination was performed using a dissecting microscope (Nikon SMZ745; Nikon, Tokyo, Japan) and an optical microscope (Olympus BX43; Olympus, Tokyo, Japan). Microphotographs were taken with the aid of a digital camera Nikon Digital Sight 1000.

Morphological examination was primarily concentrated on the appendage of ventral median scales and median band on the dorsal surface of thalli, which have proved to be of high taxonomic value in the sect. *Papillatae* (Zheng and Shimamura 2020, 2022a; Zheng 2022; Zheng and Long 2023; Zheng et al. 2024a). Epidermal papillae were also examined because it is one of the most important features that distinguishes subsp. *emarginata* from other taxa of Marchantiaceae (Bischler‐Causse 1989).

Preliminary observation was conducted on the appearance of the thalli to confirm that each specimen contains only one species. Specimens with mixed plants were not included in the present study. All examined appendages of ventral median scales were removed from the same thallus. Appendage‐removed thalli were directly used for the observation of median band on the dorsal surface of thalli.

## 3 Results and Discussion

### 3.1 Historical Account

*Marchantia emarginata* was originally published by Reinwardt, von Blume, and Nees von Esenbeck (1824) to represent a Javanese species of *Marchantia* L. with “radiis feminei emarginatis” (emarginate female rays). Gottsche, Lindenberg, and Nees von Esenbek (1846) first attempted to introduce an intraspecific classification to this species by providing a nomenclaturally invalid name *M. emarginata* f. *minor* Gottsche, Lindenb. & Nees (Art. 38.1, Turland et al. 2018). Schiffner (1893) researched exotic liverworts (Brazil and Indonesia) with special focus on the genus *Marchantia* in Lindenberg's Herbarium, and invalidly published “ *M. emarginata* β. *leucolepis*” as a synonym of *M. palmata* Reinw., Blume & Nees (Art. 36.1, Turland et al. 2018). A large taxonomic input for *M. emarginata* was made by Schiffner (1898) who created three varieties ( *M. emarginata* var. *longepedunculata* Schiffn., var. *major* Schiffn., and var. *multiradia* Schiffn.) and two forms ( *M. emarginata* f. *intermedia* Schiffn., and var. *major* f. *thermarum* Schiffn.) based on the ecological preference and morphology of thalli (shape, size, median band), ventral scales (size, color), archegoniophores (length of stalk), and female receptacles (number and apical shape of rays). At the same time, numerous taxa showing morphological similarity to *M. emarginata* have been also described (e.g., Reinwardt et al. 1824; Burgeff 1943; Stephani 1899; Bonner 1953). However, unfortunately, the studies mentioned above were primarily focused on describing novel species rather than comprehensively reviewing their taxonomic interrelationships.

In 1989, Bischler conducted a thorough character analysis on the Asian and Oceanian taxa related to *Marchantia emarginata* and rearranged this species into three subspecies with a total of approximately 30 heterogenic synonyms. In the same year, Bischler‐Causse (1989) revised *Marchantia* in Asia and Oceania, and provided additional illustrations and detailed taxonomic notes for the three subspecies. This subspecific treatment (Bischler 1989; Bischler‐Causse 1989) has been widely accepted in most subsequent studies involving relevant *Marchantia* taxa (e.g., Furuki 2002; Singh and Singh 2013), although some have not specified the identification at the subspecific level (Ho 2013; Siregar, Aritanti, and Tjitrosoedirdjo 2013; Ruklani, Rubasinghe, and Long 2015). Bischler and Piippo (1991) studied the genus *Marchantia* in the Huon Peninsula of Papua New Guinea and clearly noticed the variation among *M. emarginata* , which may support the existence of two different forms in this species. Later, Bischler‐Causse (1993) synonymized *M. convoluta* C. Gao & K.C. Chang to *M. emargnata*. Although recent progresses (Zheng and Shimamura 2022a, 2022b; Zheng and Long 2023; Zheng et al. 2024a) have made minor adjustments to some synonyms of *M. emarginata* , these studies are still largely based on the treatment of Bischler (1989).

To summarize, the subspecific classification of *Marchantia emarginata* established and amended by Bischler (1989) and Bischler‐Causse (1989) has been widely accepted, although some details warrant careful review.

### 3.2 On the Subspecific Classification

**FIGURE 2:**
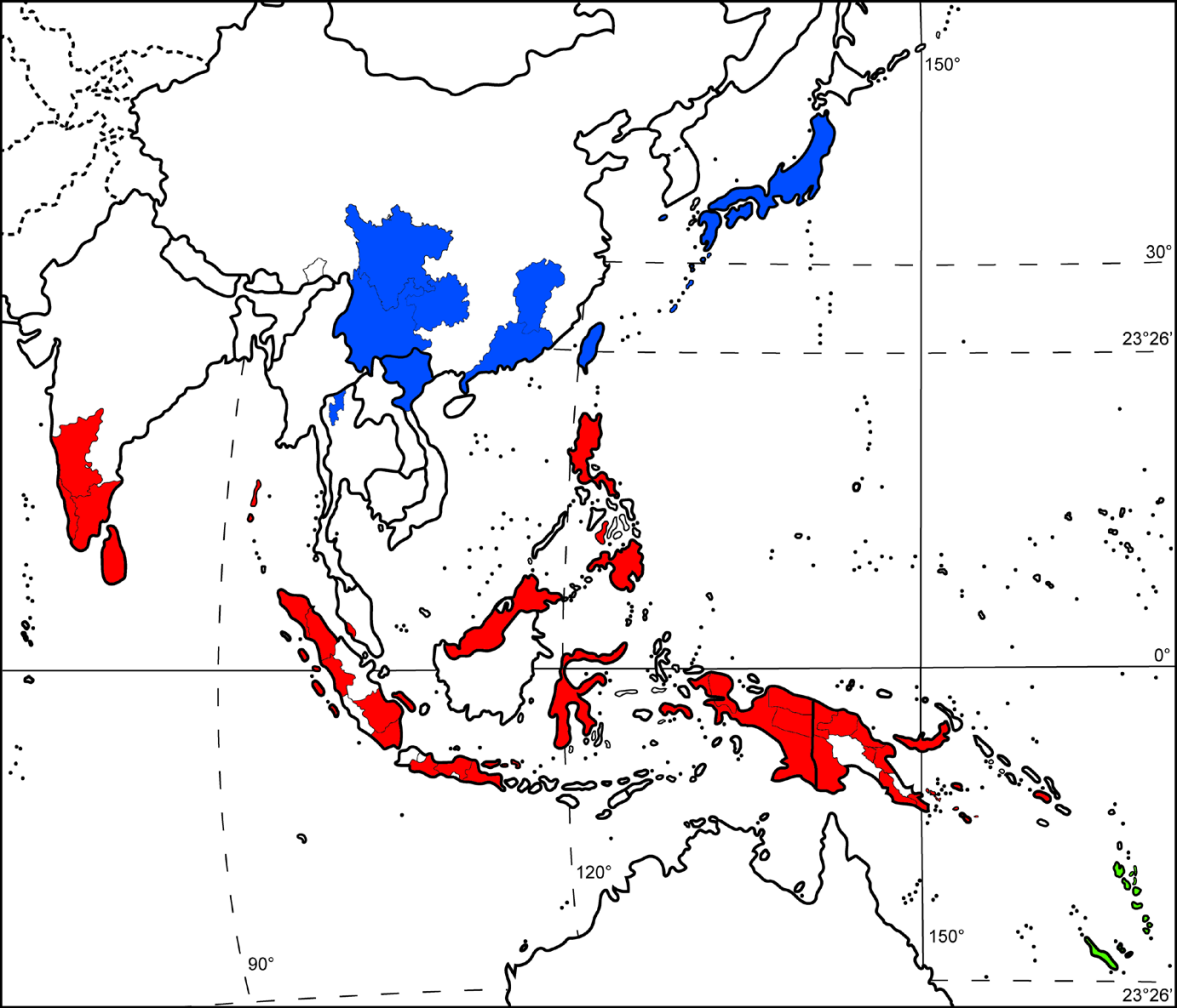
The geographical distribution of the three subspecies of *Marchantia emraginata* Reinw., Blume & Nees summarized from Bischler (1989) and Bischler‐Causse (1989). Blue, red and green indicated the distribution subsp. *tosana* (Steph.) Bischl., subsp. *emarginata* and subsp. *lecordiana* (Steph.) Bischl., respectively.

As previously mentioned, *Marchantia emarginata* is widely distributed in Asia and Oceania, and ascribed broad morphological definition. Bischler (1989) creatively divided this species into three subspecies with geographical segregation: subsp. *emarginata* for tropical Southeast Asia and parts of Oceania (Papua New Guinea and Solomon), subsp. *tosana* (now called as subsp. *cuneiloba* (Steph.) T.X. Zheng & M. Shimamura) for East Asia, and subsp. *lecordiana* (Steph.) Bischl. for Oceania (New Caledonia and Vanuatu; Figure 2). Following to this rule, each subspecies was assigned with synonyms originally described from corresponding region (Bischler 1987, 1989; Bischler‐Causse 1993). It is noted that the recent report of subsp. *emarginata* from India (Singh and Singh 2013) was not included here because the present discussion needed to be as consistent as possible with Bischler's (Bischler–Cuasse's) original conception. My specimen examination and perusal of literature reveal a challenging view that this geography‐based classification and taxonomic status of subsp. *emarginata* and subsp. *lecordiana* are problematic, as demonstrated for *M. emarginata* subsp. *tosana* in Japan by Zheng and Shimamura (2022b).

**FIGURE 3:**
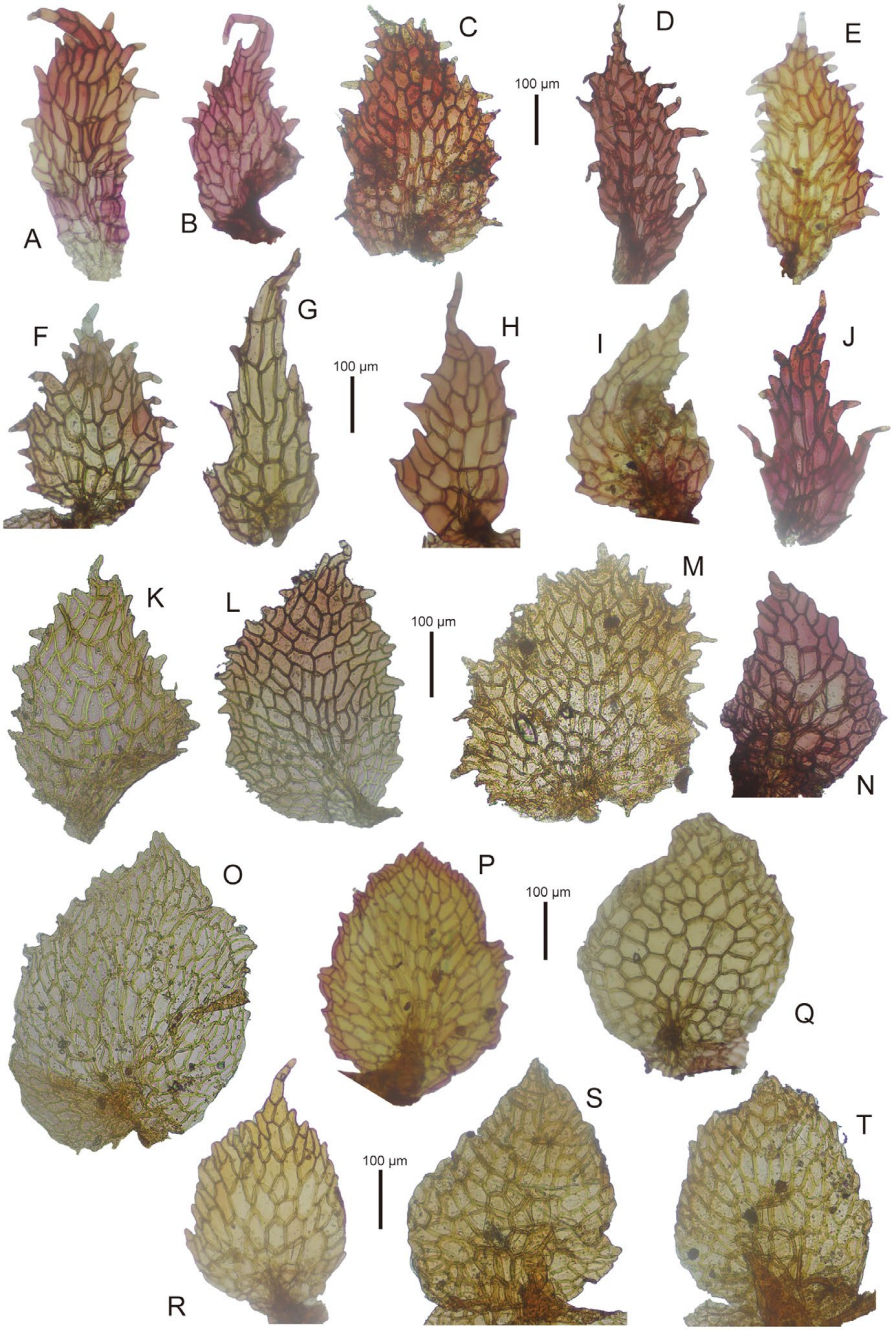
Appendages of median scales of *Marchantia emraginata* subsp. *emarginata*. (A) *J. L. De Sloover 42703* (CBG). (B) *H. Streimann & E. Tamba 11747* (CBG*). (C) *S. J. van Oostsroom 13198* (L*). (D) *R. D. Hoogland 11475* (CANB*). (E) *H. Streimann 13514* (CBG*). (F) *H. Streimann 25259* (CBG*). (G) *H. Streimann 17014* (CBG*). (H) *H. Streimann 19936* (CBG*). (I) *H. Streimann & T. Umba 11222* (CBG*). (J) *Wilde & Wilde‐Duyfjes 12241* (L*). (K) *Korthals s.n*. (L*). (L) *Anonymous s.n*. (L*4460434). (M) *Korthals s.n*. (L* 4460470). (N) *H. Streimann 12,475* (CBG*). (O) *W. Meijer B7445* (L*). (P) *R. van der Wijk 1605* (L*). (Q) *L. Hoffmann 89‐285* (CBG). (R) *G. H. S. Wood 1285* (L*). (S, T) *Anonymous s.n*. (L*4460462).

**FIGURE 4:**
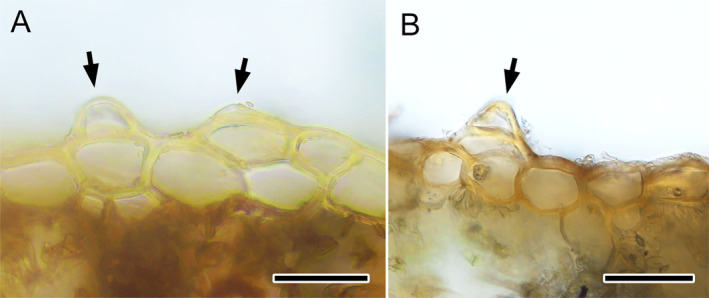
Epidermal papillae in *Marchantia emraginata* subsp. *emarginata*. (A) *M. Jacobs B798* (L). (B) *van Zanten 875* (NICH*). Arrows: Epidermal papillae. Scales = 50 μm.

Contrary to the “accepted” confidence level adopted by Söderström et al. (2016), *Marchantia emarginata* subsp. *emarginata* appears to be a poorly defined taxon. First, *M. emarginata* subsp. *emarginata* shows significant morphological diversity in the appendages of the ventral median scales among the specimens identified by Bischler (Figure 3). Some appendages are narrower and toothed (Figure 3A–J), matching the characteristics illustrated and discussed by Bischler (1989: figure 5) and Bischler‐Causse (1989: figure 55), while others (Figure 3K–T) tend to be broader and less toothed, resembling *M. papillata* subsp. *grossibarba* (Steph.) Bischl. (Bischler‐Causse 1989; Zheng and Shimamura 2020, 2022a, 2022b). Such morphological diversity also extends to the median band of the thalli (data not shown), which was considered absent in subsp. *emarginata* but distinct in *M. papillata* (Bischler‐Causse 1989). However, continuous bands are found not only in some ordinary specimens of subsp. *emarginata* (e.g., *W. Meijer 7207*, *Streimann & Kairo 35892*, and *Wilde & Wilde‐Duyfjes 12241*) but also in part of its conspecific taxa (e.g., *M. emarginata* var. *multiradia* and *M. stenolepida* Herz. ex Burgeff; Burgeff 1943). Since these materials of subsp. *emarginata* were identified by Bischler herself, who explicitly mentioned that *M. papillata* and subsp. *emarginata* do not share overlapping geographical distribution (Bischler 1989; Bischler‐Causse 1989). The morphological boundary between *M. emarginata* subsp. *emarginata* and *M. papillata* is thus not clearly recognized, as pointed out by Zheng and Shimamura (2022b) between subsp. *tosana* and *M. papillata* . An incomplete understanding of subsp. *emarginata* is also reflected in epidermal papillae, which was considered the most distinctive character of this subspecies (Bischler 1989; Bischler‐Causse 1989). My observation, however, suggested that epidermal papillae are not a common feature as it was found in only two specimens (*M. Jacobs B798* (L), *van Zanten 875* (NICH*); Figure 4). This character might be an accidental rather than a stable feature.

**FIGURE 5:**
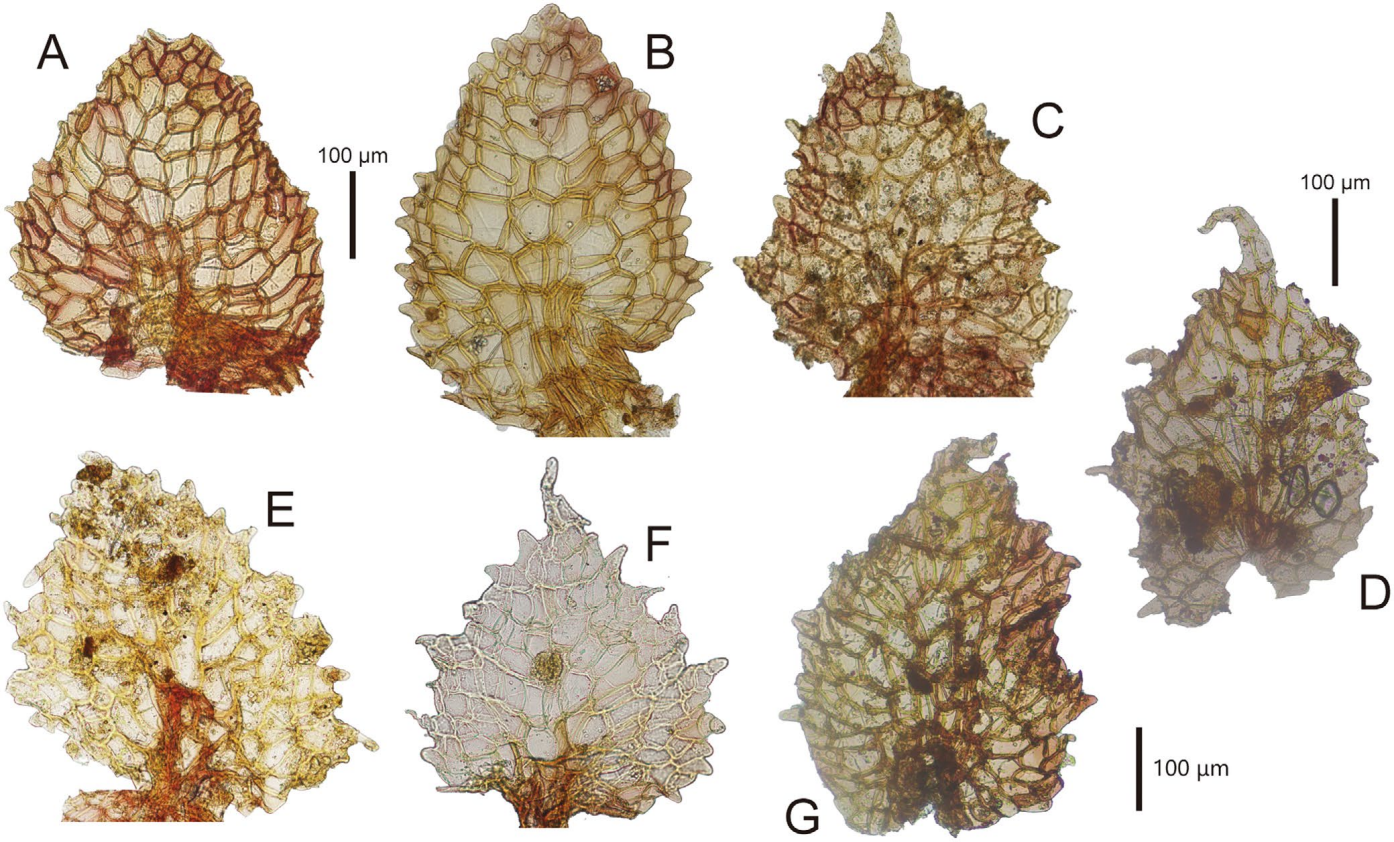
Appendages of median scales of *Marchantia emraginata* subsp. *lecordiana* (Steph.) Bischl. (A) *Lecord s.n*. (G* 43729). (B, C) *Lecord s.n*. (G* 43730). (D) *Le Rat s.n*. (L*). (E, F) *Sugimura 1418* (TNS). (G) *I. Thériot 24* (L*).

Unlike *Marchantia emarginata* subsp. *emarginata*, subsp. *lecordiana* may represent a misrecognition from my point of view. Since its formal inception, this subspecies has been challenging to access taxonomically, owing to its extremely limited geographical distribution and available materials. I examined the appendages of the ventral median scales of types and some ordinary specimens of subsp. *lecordiana* (Figure 5). The appendages of its type specimens (Figure 5A–C) showed a strong resemblance to *M. papillata* (Zheng and Shimamura 2020, 2022a, 2022b). In addition, the only color photograph of this subspecies was provided by Brown (2011, figure 132), in which a blackish median band can be vaguely seen from the dorsal surface of the thalli. Unfortunately, this specimen was unavailable for the present study, and thus I am not able to propose a conclusion on this aspect. Nevertheless, *M. emarginata* subsp. *lecordiana* also shows close resemblance with *M. papillata* in the morphology of the ventral appendages because this subspecies has no synonyms (Bischler 1989), and its morphological definition is entirely based on the type specimens examined in this study.

Furthermore, based on the incongruencies noted above, it can be concluded that the subspecific treatment of *Marchantia emarginata* is likely based on geography rather than morphology. Specifically, to establish and maintain a stable taxonomic status for each subspecies, the morphological diversity existing within their respective geographical ranges was ignored to some extent.

The taxonomy of *M. emarginata* is much complex than currently recognized, as this study finds numerous intermediates that could not be accurately identified. This geography‐based classification indeed facilitates rapid identification of this widely distributed and morphologically complicated species. However, it also introduces a side‐effect that each subspecies can be identified based on their collection site rather than exterior characteristics.

In summary, high morphological diversity was newly noticed in some key traits from the two subspecies of *Marchantia emarginata* , indicating that the boundary between *M. emarginata* and *M. papillata* is unclear and requires further review.

### 3.3 Experience on Morphological Examination

In this study, the morphological examination of *Marchantia emarginata* focused primarily on the appendages of the ventral scales and the median band of the thalli. For aged specimens, the former character seems to be reliable because they do not vary significantly after dehydration. However, there are slight differences between vegetative and sexual plants. This character tends to be essentially uniform in sterile specimens but become narrower as it gets closer to the base of the archegoniophore stalks in fertile plants. A similar trend was also observed in specimens with unelevated female receptacles. In other words, the ventral median appendages near the archegoniophore stalks are of less taxonomic value.

As for the median band of thalli, Zheng and Shimamura (2022b, figure 3) identified two types (continuous and discontinuous) by giving clear color micrographs of fresh plants. In the present study, plants with discontinuous bands were rarely observed, which may be due to long‐term desiccation. In addition, continuous band needs special attention. This character, caused by the thallus folding inward along the midline after drying, or the blackening of the middle part of the thallus due to prolonged water immersion, was observed in the present study and should not be considered taxonomically significant as they are no more than variation led by experimental conditions. This might explain why Bischler‐Causse (1989) sometimes noted a blackish median band in both subsp. *emarginata* and subsp. *tosana*. The continuous band is actually caused by “region without air‐chamber,” as noted and illustrated by Zheng and Shimamura (2020).

### 3.4 Miscellaneous Thought

Current treatment and taxonomic comprehension of sect. *Papillatae* are far from straightforward. Based on the above evidence, the interspecific boundary between *Marchantia emarginata* and *M. papillata* appears ambiguous and poorly recognized, which may have led to the misarrangement of synonyms (e.g., *M. tosana* Steph. and *M. tosayamensis* Steph.; Zheng and Shimamura 2022b). Additionally, since sect. *Papillatae* includes taxa distributed in South America ( *Marchantia papillata* subsp. *papillata*) and Africa ( *Marchantia debilis* Goebel), and almost all taxa in the section have been assigned their respective synonyms, it is highly possible that the morphological boundaries of the species within this section have also been improperly recognized and described. To address this issue, a comprehensive review of *M. emarginata* and its congeners, including synonyms, is necessary, rather than conducting regional revisions. Given the presence of many morphological intermediates, a classification from a purely morphological perspective is not feasible. Thus, it is recommended to perform molecular phylogenetic analysis, deduce the taxonomically informative characteristics of each molecular group, and then arrange the corresponding synonyms. In fact, Zheng and Shimamura (2022b) has demonstrated the feasibility of such integrative study for the sect. *Papillatae*, especially for clarifying the delimitation between *M. emarginata* and *M. papillata* .

The conclusions of this study, were obtained based on morphological examinations and a review of existing literature, which may vary with the introduction of molecular phylogeny in the future. Nevertheless, the existence of taxonomic issues within the *Marchantia emarginata* complex and its related taxa has been clearly demonstrated.

## Author Contributions

**Tian‐Xiong Zheng:** conceptualization (equal), data curation (equal), formal analysis (equal), funding acquisition (equal), investigation (equal), methodology (equal), project administration (equal), resources (equal), software (equal), supervision (equal), validation (equal), visualization (equal), writing – original draft (equal), writing – review and editing (equal).

## Conflicts of Interest

The author declares no conflicts of interest.

## Data Availability

All data can be found in the main text.

## Appendix A

**Representative specimens examined**. Herbarium accession/barcode number was additionally given for the specimens without specific collection numbers. ***Marchantia emarginata*** subsp. ***emarginata***: borneo: *Korthals s.n*. (L 4460404, 4460406), *Korthals s.n*. (L* 0793371), *Korthals s.n*. (L* 4460469), *Korthals s.n*. (L* 4460470). indonesia: *Waitz s.n*. (L*); Ambon, *Zippelius s.n*. (L*); Bali Island, near rock tombs, June 1929, *W. A. Setchell s.n*. (NY*); Celebes, Minahassa, 1921–1922, *B. Berends ten Kate s.n*. (L*); Ceram, 16–25 Apr. 1938, *P. J. Eyma 3279* (L*); Flores, Ruteng, near Mission station, 1050 m alt., 12 June 1975, *J. F. Veldkamp 6978A* (L*); Java, *H. O. Forbes 1176* (L*), *S. J. van Oostsroom 12886* (L*), *S. J. van Oostsroom 13198* (L*), *S. J. van Oostsroom 13195* (L*), *van Steenis s.n*. (L* 4460422), *Thunberg s.n*. (L*), *Zippelius s.n*. (L*), *Anonymous s.n*. (L* 4460384), *Anonymous s.n*. (L* 4460385), *Anonymous s.n*. (L* 4460434), *Anonymous s.n*. (L* 4460461), *Anonymous s.n*. (L* 4460462), 1804, *H. Zollinger s.n*. (L*), Bandang, 1805, *Anonymous s.n*. (L* 4460435), Bandung, *A. Zollinger 340* (L*), Bandung, Lembang, *ca*. 1200 m alt., 24 July 1949, *A. J. Luitingh s.n*. (L*), *A. J. Luitingh 49‐7‐4* (L*), Buitenzorg, copiose, 1 May 1897, *E. Nyman s.n*. (NY*), Buitenzorg, Res. Batavia, *ca*. 250 m alt., June 1930, *F. Verdoorn 132* (NY*), Kalangan, *Junghuhu s.n*. (L), Kandang Badak, 2150 m alt., 19 Mar. 1952, *R. van der Wijk 498* (L*), G. Pangerango, *Blume s.n*. (L*), *Anonymous s.n*. (L* 4460476), lac de Telaga bodas sur Garoet, talus terreux du chemin, 1350 m alt., 25 Oct. 1904, *B. P. G. Hochreutiner 2174* (L*), Goenoeng Haloe, Res. Priangan, May 1930, *ca*. 1200 m alt., *F. Verdoorn s.n*. (NY*), Limbang, *Korthals s.n*. (L*), Manokwari, 13 June 1956, *C. Kalkman 3529* (L*), Maribaja, 1200–1300 m alt., *R. van der Wijk 750* (L*), Modjokerto, *Anonymous s.n*. (L* 4460460), Salak, 16 Apr. 1897, *E. Nyman s.n*. (L*), Dec. 1855, *H. Zollinger 340* (L*), Telaga Warna, 16 Mar. 1952, *R. van der Wijk 308c* (L*), Tjibodas, 1400 m alt., Feb. 1985, *Hallier 604* (L*), Tjibodas, *ca*. 1420 m alt., 28 Apr. 1894, *V. Schiffner 42* (L*, NY*), Tjibodas, waterfall Tjiwalen, 1500 m alt., 29 Feb. 1952, *R. van der Wijk 17g* (L*); Medinie, *Junghuhn s.n*. (L); Near Tjibodas, *Hasskarl s.n*. (L*); New Guinea, Sabronsamon (zuidwest van het Sentani‐meer), *ca*. 180 m alt., 13 Aug. 1957, *C. Kalkman BW6212* (L*), Jayawijaya, *van Zanten 875* (NICH*); West Java, near Buitenzorg, Nov. 1859, *Kurz s.n*. (L), Bogor, 20 Jan. 1860, *Kurz s.n*. (L), *ca*. 260 m alt., 14 Mar. 1894, *V. Schiffner 33* (L*), Lembang, Margahaju, 8 Apr. 1972, *A. P. Everaarts 52* (L*), Tjiburrum, auf einem Waldpfad, 1700 m alt., *Meijer 1952* (JE*); Sumatra, *Goebel 1925* (JE*), *Kausha s.n*. (JE*), Berastagi, 1928, *C. Hamel & R. S. Toroes 466* (NY*), between Sumato and Bukit Tinggi, Karbouwengat, 4 June 1952, *R. van der Wijk 1307* (L*), *R. van der Wijk 1335* (L*), Brastagi, Pasangrahan, 18 June 1952, *R. van der Wijk 1605* (L*), Koloh, 28 May 1952, *R. van der Wijk 1230* (L*), Medan Kajoo Aro, July 1931, *F. Wysling 163* (L*), Padang Pandjang, 1 June 1952, *R. van der Wijk 1272* (L*), *R. van der Wijk 1278* (L*), *R. van der Wijk 1279* (L*), Padang Pandjang, grounds of Sekolah Guru Atas, 8 June 1952, *R. van der Wijk 1391* (L*), Petani, 940 m alt., 18 June 1952, *R. van der Wijk 1568* (L*), North Sumatra Province, Ketambe, valley of Lau Alas, near tributary of Lau Ketambe, *ca*. 35 km NW of Kutatjane, 200–400 m alt., 21 May 1972, *Wilde & Wilde‐Duyfjes 12241* (CBG*, L*), West Sumatra Prov., 1200 m alt., Oct. 1949, *W. C. Verboom s.n*. (L*), Karbau canyon near Bukit tinggi, 900 m alt., 8 Mar. 1956, *W. Meijer 6428* (L*), Sikoengkang, 27 May 1952, *R. van der Wijk 1215* (L*), *R. van der Wijk 1223* (L*); Panti Tjoobadak, 700 m alt., *Bünnemeijer 30A* (L*); Purba (cote N du lac Toba, entre Merek et Pematangsiantar), parc du village d l'ancien roi, 1250 m alt., 20 Nov. 1989, *L. Hoffmann 89‐285* (CBG); Lesser Sunda Islands, *J. A. J. Verheijen 2963* (L*); West Sumatra, *W. Meijer 7207* (L*), Mt. Merapi, 1500 m alt., 12 June 1955, *W. Meijer B7445* (L*). malaysia: Sarawak, Nanga Mujong, 9 Aug. 1954, *W. M. A. Brooke 8978* (L*), State Perak, by path leading to Taiping Swimming Club, 2 miles East of Taiping, 27 Dec. 1953, *G. H. S. Wood 1285* (L*). papua new guinea: Central Prov., between head of Goldie River & Ower's Corner, 40 km ENE of Port Moresby, 500 m alt., 9 Feb. 1981, *H. Streimann & E. K. Naomi 14845* (CBG*), Tapini Subdistrict, Tapini, 29 Apr. 1971, *M. J. E. Coode 3792* (L*); East Sepik Prov., Kairuru Island, 600 m alt., 10 Nov. 1981, *O. W. Borrell 81‐15* (JE*), *O. W. Borrell 81‐18* (CBG*); Madang Prov., ile volcanique de Manam, entre le village de Waris et le Sommet de l'ile, 300 m alt., 27 Mar. 1987, *J. L. De Sloover 42703* (CBG); Morobe Prov., about 1.5 miles east of Bulolo, 800–1000 m alt., 7 Dec. 1974, *G. A. Shea 6570* (CBG*), Aseki‐Madamna Track, 1 km SW of Aseki, 1350 m alt., 23 Jan. 1981, *H. Streimann 12475* (CBG*), Boana‐Markham Valley Road, 4 km SW of Boana, 880 m alt., 10 Nov. 1982, *H. Streimann 25767* (CBG*), Busu River, 20 km NNW of Lae, 100 m alt., 9 June 1982, *H. Streimann 19929* (CBG*), *H. Streimann 19936* (CBG*), Crooked Creek, 4 km of Bulolo, 800 m alt., 20 May 1982, *H. Streimann 19760* (CBG*), Edie Creek Road, above Wau, 21 May 1963, *P. van Royen 16281* (L*), Gabensis Creek, 29 km W of Lae, 120 m alt., 14 Jan. 1981, *H. Streimann & T. Umba 11222* (CBG*), Kaisinik Village, 7 km SE of Wau, 1100 m alt., 29 Jan. 1981, *H. Streimann 13738* (CBG*), *H. Streimann 13742* (CBG*), Koke Village, 3 km SE of Aseki, 1500 m alt., 20 Jan. 1981, *H. Streimann & E. Tamba 11747* (CBG*), Near Nauti Village, Upper Watut River, 15 km SW of Bulolo, 830 m alt., 28 Jan. 1981, *H. Streimann 13514* (CBG*), *H. Streimann 13517* (CBG*), Road 3, Rifle Range L. A., 6 km SW of Bulolo, 1200 m alt., 25 Feb. 1982, *H. Streimann 17014* (CBG*), Sangkwep Logging area NE of Lae, *ca*. 200 m alt., *A. Touw 14668* (L*), Tauri River, Menyamya, 1200 m alt., 29 Apr. 1982, *H. Streimann 18920* (CBG*), Upper Watut River at Nauti Village, 14 km SW of Bulolo, 1200 m alt., 14 Oct. 1982, *H. Streimann 25254* (CBG*), *H. Streimann 25259* (CBG*), *H. Streimann 25262* (CBG*, JE*, NICH*), *H. Streimann 25264* (CBG*), *H. Streimann 25268* (CBG*), Water Resources Camp, Taiak, Middle Watut River, 12 km SSW of Mumeng, 600 m alt., 29 Dec. 1982, *H. Streimann 33024* (CBG*), Watuma, Menyamya Sub‐dist., 4000 ft alt., 10 May 1968, *H. Streimann & A. Kairo 35892* (CBG*, L*); Sepik Dist., Aitape Subdist., along Pieni River, near Walwali village, Susuku, 30 m alt., 20 June 1961, *P. J. Darbyshire 7972* (CANB, L*). philippines: Mindanao, Camiguin, March–April, 1912, *M. Ramos 2‐327* (NY*), Zambales, Mt. Pinatubo‐villar, 400 m alt., 6 Sep. 1948, *R. B. Fose 4764* (L); Leyte, roadside of Babybay Mt. Road, Streamlet, 10 Aug. 1957, *G. Frehne 56725* (L*); Luzon, May 1909, *M. Ramos 8281* (L*), May 1911, *C. B. Robinson 14131* (L*), 1968, *M. Jacobs B798* (CBG, L*), Albay Prov., Mayon Volcano, May–June 1953, *D. R. Mendoza 20643‐3* (L*), 31 May 1953, *D. R. Mendoza 56725* (L*), Apayao Subprov., Mt. Magnas, June 1953, *G. E. Edaño 20643‐3* (L*), *G. E. Edaño 56725* (L*), Benguet Subprov., May–June 1904, *P. T. Barnes 20191* (NY*), May 1911, *E. D. Merrill 7885* (JE*, NY*), *E. D. Merrill 7890* (NY*), Cavito, 1906, *Mangubat 1281* (L*), Laguna, Mar. 1910, *O. W. Calyin 336* (NY*), Lutab to Kabayan, June 1909, *R. C. McGregor 86219‐2* (NY*); Mountain Prov., Ifugao, Banaue, Bayninan, 27 Nov. 1962, *D. R. Mendoza & Buwaya 73924‐2* (L*), Mt. Tabayoc, 1968, *M. Jacobs B730* (CBG*, L*), Pampanga Prov., Mt. Pinatubo, Camp Stotsenburg, May 1927, *A. D. E. Elmer 22229* (L*), *A. D. E. Elmer 22262* (L*), Tayabas Prov., May 1907, *A. D. E. Elmer 7646A* (L*, NY*); Camaguin de Mindanao, *M. Ramos 14884* (L*). salomon: N. W. Guadalcanal, 10 m alt., 1965, *Dennies s.n*. (JE*), Guadalcanal, Honiara, Botanical Garden at base of damp loam‐bank in secondary forest, 1968, *van Zanten 68‐2319* (JE*). sri lanka: Central Prov., Kandy Dist., Murutenne (*ca*. 2 miles W of Norton Bridge), 900 m alt., 3 Mar. 1969, *R. D. Hoogland 11475* (CANB*, L*, JE*), communicavit Pasupathy via Huneck, 400 m alt., 1976, *Anonymous s.n*. (JE*‐H1597); Haputale, 6 Aug. 1977, *M. Onraedt s.n*. (JE*). ***Marchantia emarginata*** subsp. ***cuneiloba***: japan: Shizuoka Prefecture, Shizuoka City, Suruga District, Hirasawa, *T.‐X. Zheng 1547* (HIRO); Hiroshima Pref., Higashi‐Hiroshima City, Hara, *T.‐X. Zheng 1506* (HIRO); Wakayama Pref.: Higashimuro County, Matsune, *T.‐X. Zheng 1536* (HIRO); Tottori Pref., Saihaku County, Houkimizoguchi, Y. *Horikawa 3863* (HIRO); Saga Pref.: Karatsu City, Ouchi‐cho, Ikisa, *T.‐X. Zheng 1145* (HIRO); Nagasaki Pref.: Unzen City, Obara‐cho, Minamihon‐machi, *T.‐X. Zheng 1155* (HIRO); Kumamoto Pref.: Hitoyoshi City, Nishiotsuka‐machi, *T.‐X. Zheng 1105* (HIRO); Kagoshima Pref.: Kanoya City, Nishihara, *T.‐X. Zheng 1075* (HIRO), Kumage Co., Yakushima‐cho, *Y. Horikawa 11873* (HIRO); Okinawa Pref.: Ishigaki City, Mezato, *T.‐X. Zheng 792* (HIRO). Naha City, Shurisueyoshicho, *T. Amano 8112* (NICH*). ***Marchantia emarginata*** subsp. ***lecordiana***: vanuatu: Efate Island, Pang Pang, 20 m alt., 3 Oct. 1997, *Sugimura 1418* (TNS); Butmas, 540 m alt., 23 Oct. 1997, *Sugimura 1741* (TNS); new caledonia: *I. Thériot 24* (L*), 700–800 m alt., June 1905, *Le Rat s.n*. (L*), Dogny, 1072 m alt., 1909, *Le Rat s.n*. (L*), Ouraї, *Lecord s.n*. (G* 43729, 43730). ***Marchantia papillata*** subsp. ***grossibarba***: china: Sichuan Prov.: Yanbian County, *M.‐Z. Wang 20126* (PE); Xizang, Mêdog Cou., ad vias, 13 Sep. 1974, *J.‐W. Zhang M7417* (IFP); Mêdog Xian, ad ripas, 18 Aug 1974, *C. S. Kun 53* (IFP); Yunnan Prov., Mengla Co., Menglun, *P.‐C. Wu 21708* (PE), bois de Santcha‐ho, June 1887, *P. J. M. Delavay s.n*. (G* 43749). india: Pubjab, *Koelz 4420* (NICH*); Uttar Pradesh, *Gollan 4585* (G*); West Bengal, *Hartless 3324* (G). myanmar: *Dickason 7325* (NICH*). japan: Saitama Pref.: Saitama City, Nakao, *T.‐X. Zheng 1484* (HIRO). Chiba Pref., Kamogawa City, Yomogi, *T.‐X. Zheng 328* (HIRO); Tokyo: Higashikurume City, Gakuen‐cho, *Kifuji 375*; Ishikawa Pref.: Hakusan City, Chuugu, *T.‐X. Zheng 1615* (HIRO); Gifu Pref.: Yamagata City, Kanzaki, *T.‐X. Zheng 412* (HIRO); Shiga Pref.: Maibara City, Samegai, *T.‐X. Zheng 1523* (HIRO); Hyogo Pref.: Asago City, Ikuno‐cho, *T.‐X. Zheng 1498* (HIRO); Yamaguchi Pref.: Iwakuni City, Oze, *Katsui 444* (HIRO), Mine City, Shuhou‐cho, *Shiomi 19* (HIRO), *T.‐X. Zheng 1157* (HIRO); Tokushima Pref.: Myouzai Co., Kamibun, *T.‐X. Zheng 1251* (HIRO); Kochi Pref.: Kochi City, Tosayamakuwao, *T.‐X. Zheng 1228* (HIRO).
